# Modeling the cost-effectiveness of mass screening and treatment for reducing *Plasmodium falciparum* malaria burden

**DOI:** 10.1186/1475-2875-11-S1-P19

**Published:** 2012-10-15

**Authors:** Valerie Crowell, Olivier JT Briëf, Diggory Hardy, Nakul Chitnis, Nicolas Maire, Aurelio Di Pasguale, Thomas A Smith

**Affiliations:** 1 Department of Epidemiology and Public Health, Swiss Tropical and Public Health Institute, Socinstrasse 57, P.O. Box, CH-4002 Basel, Switzerland; 2 University of Basel, Petersplatz 1, P.O. Box, CH-4003 Basel, Switzerland

## Background

Past experience and modeling suggest that, in most cases, mass treatment strategies are not likely to succeed in interrupting *Plasmodium falciparum* malaria transmission. However, this does not preclude their use to reduce disease burden. Mass screening and treatment (MSAT) is preferred to mass drug administration (MDA), as the latter involves massive over-use of drugs. This paper reports simulations of the incremental cost-effectiveness (ICER) of well-conducted MSAT campaigns as a strategy for *P.falciparum* malaria disease burden reduction in settings with varying receptivity (ability of the combined vector population in a setting to transmit disease) and access to case management.

## Materials and methods

MSAT incremental cost-effectiveness ratios (ICERs) were estimated in different sub-Saharan African settings using simulation models of the dynamics of malaria and a literature-based MSAT cost estimate. Imported infections were simulated at a rate of 2 per 1,000 population per annum. These estimates were compared to the ICERs of scaling up case management or insecticide treated net (ITN) coverage in each baseline health system, in the absence of MSAT.

## Results

MSAT averted the most episodes, and resulted in the lowest ICERs, in settings with a moderate level of disease burden. At a low pre-intervention entomological inoculation rate (EIR) of 2 infectious bites per adult per annum (ibpapa), MSAT was never more cost-effective than scaling up ITNs or case management coverage. However at pre-intervention EIRs of 20 and 50 ibpapa and ITN coverage levels of 40 or 60%, respectively, the ICER of MSAT was similar to that of scaling up ITN coverage further.

## Conclusions

In all the transmission settings considered, achieving a minimal level of ITN coverage is a “best buy”. At low transmission, MSAT probably is not worth considering. Instead, MSAT may be suitable at medium to high levels of transmission and at moderate ITN coverage. If undertaken as a burden-reducing intervention, MSAT should be continued indefinitely and should complement, not replace, case management and vector control interventions.

